# Casualties of German Army Surgeons

**Published:** 1916-04

**Authors:** 


**Casualties of German Army Surgeons.** Up to Dec. 25, 1915, 1,084 army surgeons were included in the casualty lists: 37 civilian physicians, 377 active medical officers, 373 officers of reserves, 287 assistants. Of the total, 361 were killed, 142 severely wounded, 388 less severely wounded, 102 taken prisoner, 90 missing. It will be noted that the ratio of killed, exclusive of future deaths among the wounded and of the missing, was almost exactly 1:3, which is more than the general ratio of past wars for the troops in general, and probably close to the average for the present war. The army surgeon shares to the full the risk of the soldier.

